# Defining the Protein Phosphatase 2A (PP2A) Subcomplexes That Regulate FoxO Transcription Factor Localization

**DOI:** 10.3390/cells14050342

**Published:** 2025-02-27

**Authors:** Adeline M. Luperchio, Daniel J. Salamango

**Affiliations:** Department of Microbiology, Immunology, and Molecular Genetics, UT Health Science Center, San Antonio, TX 78229, USA; luperchioa@uthscsa.edu

**Keywords:** AKT, FoxO, PI3K, PP2A, subcellular localization, transcription factor, tumor suppressors

## Abstract

The family of forkhead box O (FoxO) transcription factors regulate cellular processes involved in glucose metabolism, stress resistance, DNA damage repair, and tumor suppression. FoxO transactivation activity is tightly regulated by a complex network of signaling pathways and post-translational modifications. While it has been well established that phosphorylation promotes FoxO cytoplasmic retention and inactivation, the mechanism underlying dephosphorylation and nuclear translocation is less clear. Here, we investigate the role of protein phosphatase 2A (PP2A) in regulating this process. We demonstrate that PP2A and AMP-activated protein kinase (AMPK) combine to regulate nuclear translocation of multiple FoxO family members following inhibition of metabolic signaling or induction of oxidative stress. Moreover, chemical inhibitor studies indicate that nuclear accumulation of FoxO proteins occurs through inhibition of nuclear export as opposed to promoting nuclear import as previously speculated. Functional, genetic, and biochemical studies combine to identify the PP2A complexes that regulate FoxO nuclear translocation, and the binding motif required. Mutating the FoxO-PP2A interface to enhance or diminish PP2A binding alters nuclear translocation kinetics accordingly. Together, these studies shed light on the molecular mechanisms regulating FoxO nuclear translocation and provide insights into how FoxO regulation is integrated with metabolic and stress-related stimuli.

## 1. Introduction

**Figure 1:**
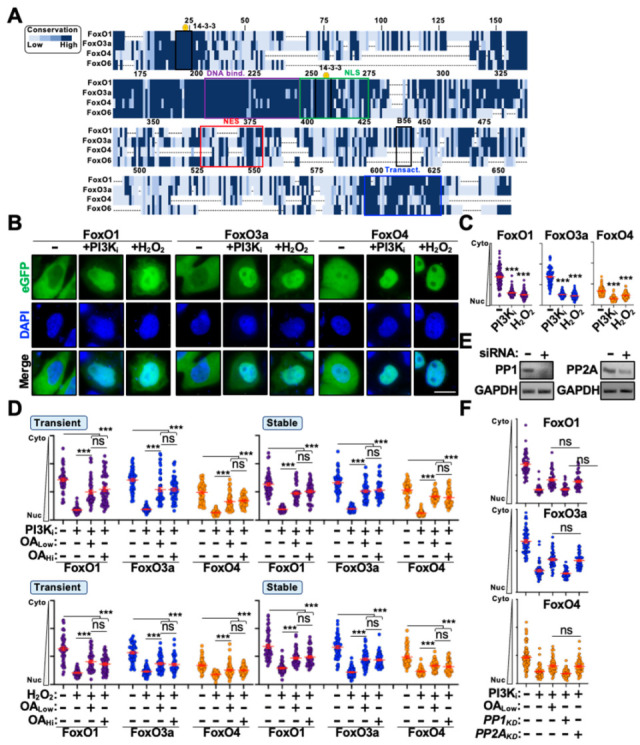
PP2A partially regulates translocation of eGFP-FoxO proteins. (**A**) Amino acid alignment of FoxO family members with shading depicting amino acid conservation amongst family members. Amino acid residues are numbered based on the FoxO1 protein sequence. (**B**) Representative live-cell fluorescence microscopy images of eGFP-FoxO proteins under unstimulated conditions or following treatment with the indicated stimulus. (**C**) Quantification of cytoplasm-to-nucleus translocation of the indicated eGFP-FoxO protein following treatment with the indicated stimulus (n = 100). (**D**) Translocation efficiency of either transiently or stably expressed eGFP-FoxO proteins under unstimulated or stimulated conditions in the presence or absence of okadaic acid treatment (n = 75). (**E**) RT-PCR results from HeLa cell lysates transiently expressing control or the indicated knockdown siRNA. (**F**) Quantification of cytoplasm-to-nucleus translocation of eGFP-FoxO stable cells transiently expressing control or siRNA following stimulation with PI3K_i_ treatment (n = 50) Scale bar = 10 μm; ns, not significant; ***, *p* < 0.001.

The evolutionarily conserved family of FoxO (Forkhead box, class O) transcription factors regulate a broad range of cellular processes that include cell cycle progression, apoptosis, DNA damage repair, and oxidative stress responses [1,2]. Humans encode four FoxO family members (FoxO1, FoxO3a, FoxO4, and FoxO6) that have highly diverse protein sequences except within functional domains where amino acid identity is relatively conserved (Figure 1). These functional domains include the family’s namesake “forkhead/winged helix” DNA binding domain, a nuclear localization sequence, a nuclear export sequence, and a C-terminal transactivation domain (Figure 1). FoxO proteins were first identified in humans at chromosomal translocation sites in multiple different cancer types. They have since been proposed to function as bona fide tumor suppressors due to their ability to regulate cell cycle and promote apoptosis [3,4]. As such, FoxO proteins have emerged as novel anti-cancer targets; however, their capacity to influence cell function is regulated by a complex network of post-translational modifications and signaling pathways that have yet to be fully characterized [4,5].

Multiple signaling pathways integrate to tightly control FoxO protein expression, subcellular localization, and DNA binding affinity [4,5]. In general, when metabolic flux is high FoxO proteins are sequestered to the cytoplasm by binding 14-3-3 dimers that occlude the nuclear import sequence [6]. When metabolic flux is low, 14-3-3 proteins disassociate to promote FoxO nuclear import and the activation of diverse transcriptional programs. The PI3K signaling cascade was the first pathway identified as a key regulator of FoxO subcellular localization and transactivation activity. Activation of PI3K from diverse stimuli induces AKT-mediated phosphorylation of conserved FoxO residues to promote 14-3-3 binding and cytoplasmic retention [7]. Further investigation has identified additional phosphorylation sites targeted by diverse signaling pathways that include stress-activated JNK (c-Jun N-terminal Kinase) signaling [8,9], nutrient deprivation-induced AMPK (AMP-activated protein kinase) signaling [10,11], serum and glucocorticoid-induced activation of SGK1 (serum and glucocorticoid-regulated kinase 1) [12,13], and growth factor-induced ERK1/2 signaling [14]. In addition to phosphorylation, acetylation [15,16], ubiquitination [17,18], and methylation [19] have been shown to regulate FoxO activity, localization, and protein stability.

As a counterbalance to AKT-mediated cytoplasmic retention, protein phosphatase 2A (PP2A) has been implicated in promoting FoxO nuclear translocation during the shift from high-to-low metabolic states [20,21]. PP2A dephosphorylates AKT target residues required for 14-3-3 engagement, which induces 14-3-3 dissociation and facilitates FoxO nuclear import [20,21]. While key phosphorylation sites and 14-3-3 binding motifs are conserved across FoxO family members, only FoxO1 and FoxO3a exhibit sensitivity to PP2A inhibition; however, only a single study has evaluated FoxO4 sensitivity to PP2A inhibition and FoxO6 is constitutively nuclear [20,21,22,23]. Moreover, PP2A functions as a heterotrimeric complex capable of forming nearly 100 unique holoenzymes that have discrete substrate interactomes [24]. These observations motivated several key questions investigated in the present study: (1) Does PP2A regulate FoxO nuclear translocation from diverse signaling inputs? (2) What are the PP2A subcomplexes that regulate FoxO nuclear translocation? (3) How does PP2A recognize FoxO proteins? The long-term goal of answering these questions is to stimulate FoxO nuclear accumulation and the activation of cell death programs as a strategy to slow tumor progression.

Here, we use a combination of genetic, pharmacologic, biochemical, and live-cell fluorescence microscopy experiments to define the PP2A subcomplexes that regulate FoxO nuclear translocation. First, we establish that PP2A and AMPK coordinate to regulate nuclear translocation of FoxO1, FoxO3a, and FoxO4 following inhibition of metabolic signaling or induction of oxidative stress. Second, we demonstrate that the nuclear accumulation of all three FoxO proteins likely involves changes to both nuclear import and nuclear export kinetics. Furthermore, these observations also indicate that FoxO proteins constitutively shuttle between the cytoplasm and nucleus under unstimulated conditions. Third, through the use of peptide inhibitors and negative protein regulators we define the PP2A subcomplexes that regulate FoxO1 and FoxO3a nuclear translocation. Lastly, using a series of rationally designed FoxO3a mutants and an *in cellulo* co-immunoprecipitation technique, we define the FoxO3a PP2A interacting motif and demonstrate that altering this motif can enhance or diminish PP2A binding affinity and alter nuclear translocation kinetics.

## 2. Materials and Methods

### 2.1. Cloning and Cell Culture

eGFP-FoxO1 and eGFP-FoxO3 expression vectors were generated by PCR amplification of cDNA generated using total RNA extracted from HeLa cell lysates. Corresponding genes were cloned into a retroviral pQCXIH expression vector using *AgeI* and *BsiWI* restriction sites that are downstream of *eGFP* to generate in-frame N-terminally tagged proteins [25]. For generating eGFP-FoxO4, a gblock was synthesized by Integrated DNA Technologies and cloned as described above. Mutant FoxO proteins were generated using overlapping PCR amplification and verified using Sanger sequencing. Constructs expressing eGFP-B56A-E and mCherry-tagged B56 peptide-inhibitors have been described previously [wild-type peptide sequence separated by serine/glycine linkers, (LPRSSTLPTIHEEELSLC)_X4_; alanine peptide sequence separated by serine/glycine linkers, (LPRSSTAPTAHAEELSLC)_x4_] [26,27]. For PME1, ARPP19, and FAM122A_29-S120C_ expression vectors, target genes were amplified from cDNA as described above and cloned into a pcDNA-5TO vector that contains an mCherry-T2A expression cassette. All PCR reactions were performed using high fidelity DNA polymerase (NEB, Ipswich, MA, USA) and all expression vectors were confirmed via Sanger sequencing.

HeLa and HEK293T cells (American Type Culture Collection, Manassas, VA, USA) were maintained in DMEM medium (Gibco cat #11-965-118) supplemented with 10% fetal bovine serum (FBS; Gibco, Gaithersburg, MD, USA) and 0.5% penicillin-streptomycin (50 units; Gibco, Gaithersburg, MD, USA). Transient transfections were performed using either 1 mg/mL polyethylenimine (PEI; Fisher #NC1014320) at a ratio of 3 μL/1 μg DNA or DharmaFECT transfection reagent. For generating stable cell lines, approximately 450,000 HEK293T cells were seeded in a 6-well plate and allowed to adhere overnight. The next day, cells were co-transfected with a VSV-G expression vector, an MLV packaging plasmid, and the corresponding genome. Medium was collected 48 h post-transfection, frozen at −80 °C for at least 6 h, and overlaid on fresh HeLa cells. HeLa cell populations were purified following hygromycin treatment 48 h post-infection.

### 2.2. Fluorescence Microscopy

Approximately 5000–8000 cells were seeded into a 96-well glass-bottom imaging plate (Ibidi #89627) and allowed to adhere overnight. For transient expression experiments, cells were transfected as described above using either 300 ng of the indicated plasmid for single transfections, or 150 ng of each plasmid for co-transfections. Cells were imaged 48 h post-transfection using a 60× oil-immersion objective on an EVOS M5000 microscope (Thermo Fisher, Waltham, MA, USA). Immediately prior to imaging, cells were washed 1× with PBS, fixed in 4% paraformaldehyde for 15 min at room temperature (Thermo Fisher, Waltham, MA, USA, #PI28906), and washed 3× with fresh PBS. For experiments using cell lines stably expressing eGFP-FoxO proteins, cells were seeded as described above and allowed to equilibrate for 48 h prior to chemical inhibitor treatments described below. For nuclei labeling, cells were treated with NucBlue live cell stain (Thermo Fisher, Waltham, MA, USA, R37605).

### 2.3. Chemical Inhibitor Treatments

To induce nuclear translocation, cells were treated with 20 μM of the PI3K inhibitor LY294002 (Sigma-Aldrich, St. Louis, MO, USA, #440202) or with 5 mM H_2_O_2_ (Thermo Fisher, Waltham, MA, USA, H325-500) for 30 min prior to imaging. For phosphatase inhibition, cells were treated with either 50 nM okadaic acid (Sigma-Aldrich, St. Louis, MO, USA, #O8010) for 5 h to inhibit PP2A, or with 1 μM okadaic acid for 1 h to inhibit both PP1 and PP2A. For AMPK inhibition, cells were treated with 5 mM dorsomorphin (Thermo Fisher, Waltham, MA, USA, #50-204-5491) overnight. To inhibit nuclear export, cells were treated with 5 ng/mL Leptomycin B (Sigma-Aldrich, St. Louis, MO, USA, #L2913) for 30 min.

### 2.4. Immunoblotting

For immunoblotting experiments, approximately 400,000 HeLa cells stably expressing the indicated eGFP-FoxO protein were seeded into a 6-well culture plate and allowed to equilibrate for roughly 72 h. Equilibrated cells were treated with vehicle or 20 μM LY294002 for 30 min prior to being washed 1× with PBS and collected in 100 μL of RIPA buffer (50 mM Tris [pH 8.0], 1 mM β-mercaptoethanol, 150 mM NaCl, 1% Triton X-100, 0.5% deoxycholate, 0.1% SDS) supplemented with a protease and phosphatase inhibitor cocktail (Thermo Fisher, Waltham, MA, USA, #78440). Cell lysates were combined with 5× sample buffer (62.5 mM Tris [pH 6.8], 20% glycerol, 5% β-mercaptoethanol, 2% SDS, 0.05% bromophenol blue), separated on an 8% SDS-PAGE gel, and transferred onto a 0.2 μm PVDF membrane (Thermo Fisher, Waltham, MA, USA, #78440). Membranes were blocked in 5% BSA in 0.01% TBST for 2 h at room temperature prior to incubation with primary antibody. Primary antibodies were diluted in 5%/TBST and gently rocked at 4 °C overnight. The next day, membranes were washed 3× with TBST, blocked in 5% BSA in TBST for 30 min at room temperature and then incubated in secondary antibody with 5% BSA in TBST for 1 h at room temperature. Membranes were washed 3× with TBST and 1× with TBS prior to a 5 min incubation with Femto chemiluminescence substrate (Thermo Fisher, Waltham, MA, USA, #34580) and being visualized on a BioRad ChemiDoc MP imaging system. Primary antibodies used were FoxO3a pS253 (1:1000, Cell Signaling Technology, Danvers, MA, USA, #9466), FoxO3a pT32 (1:1000, Cell Signaling Technology, Danvers, MA, USA, #2599), and GAPDH (1:1000, Santa Cruz, Dallas, TX, USA, #SC-32233. Secondary antibodies used were α-rabbit conjugated to HRP (1:10,000, Cell Signaling Technology, Danvers, MA, USA, #7074) and α-mouse conjugated to HRP (1:10,000, Santa Cruz, Dallas, TX, USA, #SC-525409).

### 2.5. Genetic Knockdowns and RT-PCR

For knockdown of *B56A-E* family members, previously validated shRNA constructs were introduced into a pLKO plasmid expressing mCherry in place of puromycin [26]. For knockdown of *B55A*, two targeting sequences were cloned into the pLKO-mCherry plasmid and co-expressed to achieve efficient knockdown (5′-GATCCCAGTAACAGGTCATTT-3′, 5′-CTGCAGATGATTTGCGGATTA-3′) [28]. To knockdown *PP1* and *PP2A* enzymes, siRNA SMART pools were synthesized and transiently expressed to achieve efficient knockdown (Dharmacon, Lafayette, CO, USA, PP2A: # J-003598; PP1, #J-008927; non-targeting controls, #D-001810). All experiments evaluating the genetic depletion (i.e., knockdown of target mRNA) of target factors on eGFP-FoxO localization were performed 72 h post-transfection in either wild-type HeLa cells or HeLa derivatives stably expressing the indicated eGFP-FoxO wild-type or mutant protein. To evaluate knockdown efficiency, roughly 80,000 HeLa cells were seeded into a 12-well culture plate and transiently transfected using either PEI or DharmaFECT 24 h after plating. Approximately 72 h post-transfection, cells were washed 1× in PBS and directly lysed using 500 μL TRIzol reagent (Thermo Fisher, Waltham, MA, USA, #15-596-018) and incubated at room temperature for 5 min. Lysates were transferred to a 1.5 mL Eppendorf tube and combined with 100 mL chloroform, mixed by vortexing, and incubated at room temperature for 3 min. Samples were centrifuged at maximum speed for 15 min at 4 °C. The upper phase was transferred to a fresh 1.5 mL Eppendorf tube and combined with 250 μL isopropanol for 2 h at room temperature. Samples were centrifuged at maximum speed for 15 min at 4 °C. Supernatants were decanted, and RNA pellets were washed 1× with ice cold 70% ethanol prior to air drying and resuspension in RNase free water. For cDNA synthesis, 1 μg of purified total RNA was mixed with 1 μL oligo dT primer and incubated at 65 °C for 5 min prior to the addition of reaction buffer, dNTPs, RNase inhibitor, and reverse transcriptase. The complete mixture was incubated at 42 °C for 1 h followed by a 70 °C denaturation step. For RT-PCR, 0.5 μL of cDNA was used for amplification as described above. The following primer pairs were used for amplifying the indicated gene targets: *B56A-E* primers have been published previously [26]; *GAPDH*, 5′-GAAATCCCATCACCATCTTCCAGG-3′ and 5′-CAGTAGAGGCAGGGATGATGTTC-3′; *B55A*, 5′-CAACAGGAGATAAAGGTGGTAGAG-3′ and 5′-GCTCTGGAAGGTGCTGAAGAG-3′; *PP2A*, 5′-CGTGAACGCATCACCATTCT-3′ and 5′-GCGAGAGACCACCATGTAGA-3′; *PP1* 5′-AAGTACCCCGAGAACTTCTTCC-3′ and 5′-GTAGAAACCATAGATGCGGTTGA-3′.

### 2.6. Statistical Analyses and Experimental Replicates

Quantification of eGFP-FoxO translocation was performed by first measuring nuclear eGFP pixel intensity as defined by DAPI staining and then subtracting whole cell eGFP pixel intensity to obtain a value for cytoplasmic eGFP pixel intensity. The ratio was obtained by dividing the cytoplasmic intensity by the nuclear intensity. Quantification was performed using Image J software. Statistical analyses were performed using either an unpaired two-tailed Student’s *t*-test or a one-way ANOVA in GraphPad Prism software version 10 after confirming that all data followed a normal distribution. Schematics were generated using power point. Amino acid conservation among FoxO family members was determined using Clustal Omega software and the heat map representation was generating using Excel software. All experiments were repeated at least two independent times by two different investigators.

## 3. Results

### 3.1. PP2A Only Partially Regulates FoxO Cytoplasm-to-Nucleus Translocation

To better understand the functional role of PP2A in regulating FoxO protein trafficking, we leveraged a live-cell fluorescence-based system to monitor FoxO nuclear translocation in real time. First, we generated N-terminally eGFP-tagged FoxO1, O3a, and O4 expression constructs and evaluated their responsiveness to PI3K/AKT signaling and oxidative stress. FoxO6 was excluded from these studies because under unstimulated conditions it localizes primarily to the nucleus and exhibits trafficking patterns unique from other FoxO family members [22,23]. Next, we assessed the responsiveness of eGFP-FoxO expression constructs to the inhibition of PI3K signaling (PI3K_i_; LY294002 treatment) or to the induction of oxidative stress responses, two distinct stimuli that have well-characterized roles in promoting FoxO nuclear translocation [7,29]. As depicted in Figure 1, both stimuli induced significant nuclear accumulation of all eGFP-FoxO constructs following transient expression in HeLa cells (representative images in Figure 1B, quantification in Figure 1C). Of note, under unstimulated conditions eGFP-FoxO1 and -FoxO3a localize exclusively to the cytoplasm whereas eGFP-FoxO4 is predominantly whole cell (Figure 1B), which is consistent with prior observations assessing the localization patterns of native FoxO proteins [30,31,32]. Lastly, we evaluated the responsiveness of eGFP-FoxO proteins to the inhibition of PP2A activity. We transiently expressed eGFP-FoxO constructs in HeLa cells and evaluated cytoplasm-to-nucleus shuttling in the presence of okadaic acid, which specifically inhibits PP2A at low concentrations (OA_Low_) and both PP2A and PP1 (protein phosphatase 1) at high concentrations (OA_Hi_) [33]. Interestingly, treatment with OA_Low_ only partially inhibited FoxO nuclear translocation following PI3K_i_ or H_2_O_2_ treatment (Figure 1D). To rule out the possibility of over-expression artifacts or diminished okadaic acid activity, we generated HeLa cell lines stably expressing eGFP-FoxO proteins and reevaluated nuclear translocation efficiency in the presence and absence of low and high okadaic acid concentrations. Surprisingly, modulating protein abundance or OA concentration had no discernable impact on the inhibition of nuclear translocation following stimulation (Figure 1D). Moreover, robust genetic depletion (i.e., knockdown of target mRNA) of PP2A and PP1 confirmed that the partial inhibition of nuclear translocation was specific to PP2A and that loss-of-PP2A activity only partially blocks eGFP-FoxO translocation (Figure 1E,F), which suggests at least two independent mechanisms regulate FoxO nuclear translocation.

### 3.2. AMPK and PI3K/AKT Signaling Combine to Regulate FoxO Protein Trafficking

**Figure 2:**
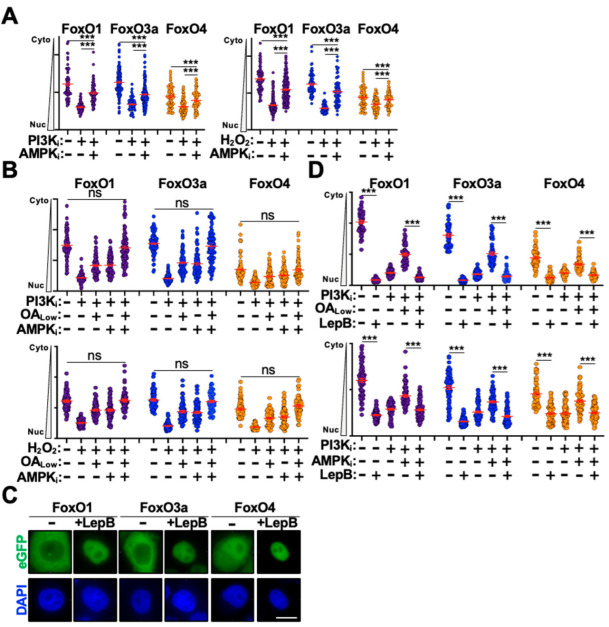
PP2A and AMPK combine to inhibit eGFP-FoxO nuclear export. (**A**) Quantification of cytoplasm-to-nucleus translocation following PIK3_i_ or H_2_O_2_ stimulation of eGFP-FoxO stable cells in the presence or absence of AMPK_i_ (n = 50). (**B**) Quantification of nuclear translocation following combination OA and AMPK_i_ treatment of eGFP-FoxO stable cells stimulated with PI3K_i_ or H_2_O_2_ (n = 50). (**C**) Representative fluorescence microscopy images of eGFP-FoxO stable cells in the presence and absence of leptomycin B. (**D**) Quantification of cytoplasm-to-nucleus translocation in eGFP-FoxO stable cells dual treated with leptomycin B and OA_Low_ (top) or AMPK_i_ (bottoms) following stimulation with PI3Ki (n = 50). Scale bar = 10 μm; ns, not significant; ***, *p* < 0.001.

Next, we wanted to define the additional mechanism(s) regulating FoxO nuclear translocation. Because inhibition of PI3K/AKT signaling and the activation of oxidative stress are both known to stimulate AMPK activity [34,35,36,37], we reasoned that AMPK signaling may also regulate FoxO nuclear translocation under these experimental conditions. Moreover, PP2A has also been shown to regulate AMPK activity in response to diverse cellular conditions [38,39,40]. Therefore, to determine if AMPK regulates FoxO nuclear translocation under these experimental conditions, we treated eGFP-FoxO stable cell lines with the AMPK inhibitor dorsomorphin (AMPK_i_) and assessed nuclear translocation following stimulation with either PI3K_i_ or H_2_O_2_. As depicted in Figure 2A, AMPK_i_ treatment only partially blocked nuclear translocation of eGFP-FoxO proteins following stimulation. We were intrigued by these observations as they mimicked the OA-mediated partial block to nuclear translocation and wondered if dual OA and AMPK_i_ treatment would fully inhibit nuclear translocation following stimulation. Indeed, co-treatment of eGFP-FoxO stable cells with both OA and AMPK_i_ resulted in full inhibition of nuclear translocation following stimulation with PI3K_i_ or H_2_O_2_, suggesting that these are the major signaling pathways that regulate FoxO nuclear translocation (Figure 2B).

The initiation of FoxO cytoplasm-to-nucleus translocation is thought to require dephosphorylation of key residues that mediate 14-3-3 engagement and cytoplasmic retention [41]. Dissociation of 14-3-3 dimers is thought to expose a nuclear-localization sequence that drives FoxO nuclear translocation; however, FoxO proteins also harbor a strong nuclear export sequence that regulates trafficking from the nucleus back to the cytoplasm [41] (Figure 1A). Emerging evidence indicates that there is a functional pool of AKT in the nucleus that regulates a wide array of cellular processes [42,43,44]. This raises the possibility that FoxO phosphorylation and inactivation might occur in the nucleus to initiate nuclear export and promote cytoplasmic retention. Moreover, the subcellular location of FoxO protein phosphorylation and dephosphorylation has yet to be conclusively established experimentally. To determine whether inhibition of PP2A and AMPK was affecting nuclear import or nuclear export kinetics, we treated eGFP-FoxO stable cells with leptomycin B to inhibit CRM1-mediated nuclear export [45]. Interestingly, leptomycin B treatment alone induced significant nuclear accumulation for all three eGFP-FoxO proteins in the absence of stimulation, suggesting that under unstimulated conditions FoxO proteins rapidly shuttle between the cytoplasm and nucleus (Figure 2C,D). Treating eGFP-FoxO stable cells with leptomycin B in combination with either OA_Low_ or AMPK_i_ blocked their inhibitory effect following stimulation with either PI3K_i_ or H_2_O_2_ (Figure 2D). Importantly, eGFP-FoxO stable cells treated with OA_Low_ or AMPK_i_ alone exhibited partial nuclear translocation inhibition following stimulation, which supports observations above and indicates that the lack of inhibition in leptomycin B treated cells is not artifactual (Figure 2D). Taken together, these observations raise the possibility that PP2A and AMPK may promote nuclear accumulation of eGFP-FoxO proteins though inhibition of nuclear export in addition to enhancing nuclear import as previous suggested.

### 3.3. Defining the PP2A Subcomplexes That Regulate FoxO Nuclear Translocation

**Figure 3:**
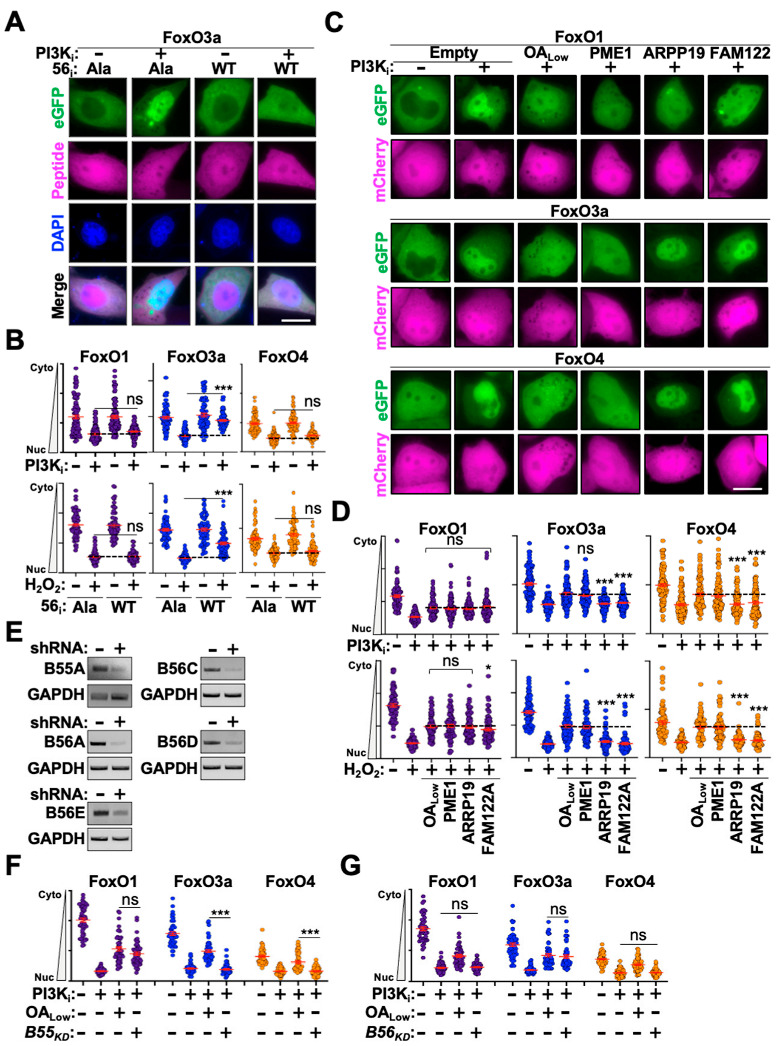
PP2A-B56 and -B55 subcomplexes regulate eGFP-FoxO nuclear translocation. (**A**) Representative fluorescence microscopy images of eGFP-FoxO3a stable cells transiently expressing wild-type and alanine B56-peptide inhibitors following PI3K_i_ treatment. (**B**) Quantification of cytoplasm-to-nucleus translocation of eGFP-FoxO stable cells transiently expressing wild-type and alanine B56-peptide inhibitors (n = 100). (**C**) Representative fluorescence microscopy images of eGFP-FoxO stable cells transiently expressing control, PME1, ARPP19, or FAM122A expression vectors following PI3K_i_ treatment. (**D**) Quantification of cytoplasm-to-nucleus translocation of eGFP-FoxO stable cells transiently expressing control, PME1, ARPP19, or FAM122A expression vectors following PI3K_i_ or H_2_O_2_ treatment (n = 75). (**E**) RT-PCR results from HeLa cell lysates transiently expressing control or the indicated knockdown construct. (**F**) Quantification of cytoplasm-to-nucleus translocation of eGFP-FoxO stable cells transiently expressing control or *B55A* knockdown constructs following stimulation with PI3K_i_ treatment (n = 50). (**G**) Quantification of cytoplasm-to-nucleus translocation of eGFP-FoxO stable cells transiently expressing control or combinatorial *B56* knockdown constructs following stimulation with PI3K_i_ treatment (n = 50). Scale bar = 10 μm; ns, not significant; *, *p* < 0.05.***, *p* < 0.001.

PP2A functions as a heterotrimeric complex comprising a phosphatase enzyme, scaffolding protein, and a regulatory subunit from one of four distinct families (B55A-D, B56A-E, PR, or STRN) [46]. The best characterized PP2A regulators are the B56A-E family which recognize cellular substrates through a canonical LxxIxE binding motif (where X indicates any amino acid) [47]. To determine if any eGFP-FoxO proteins are regulated by PP2A-B56 subcomplexes, we utilized a high-affinity peptide inhibitor that directly binds the substrate recognition region of B56 proteins to outcompete substrate interactions [27,48,49]. Transient expression of a plasmid expressing 4 tandem copies of the wild-type inhibitory peptide (LxxIxE motif) in eGFP-FoxO stable cells resulted in partial inhibition of eGFP-FoxO3a nuclear translocation following stimulation but had no effect on eGFP-FoxO1 or -FoxO4 (representative images in Figure 3A, quantification in Figure 3B). Expression of a control plasmid encoding an AxxAxA motif had no effect on translocation efficiency for any eGFP-FoxO proteins following stimulation, suggesting that FoxO3a is regulated by a PP2A-B56 complex (Figure 3A,B). Since B55 proteins are the second-best characterized family of PP2A regulators, we investigated these next. However, the substrate motif recognized by B55 proteins is highly variable [50,51,52,53,54]. Therefore, instead of directly blocking substrate recognition we instead leveraged cellular proteins known to negatively regulate PP2A activity and the activity of specific PP2A-B55 sub-complexes [55,56,57,58,59]. We generated constructs expressing PME1 (general PP2A inhibitor), ARPP19 (PP2A-B55-specific inhibitor), or FAM122A (PP2A-B55-specific inhibitor) upstream of an mCherry reporter expressed from an independent promoter. These constructs were transiently expressed in eGFP-FoxO stable cells and nuclear translocation efficiency was assessed following stimulation. As anticipated, transient expression of PME1 partially blocked nuclear translocation to the same degree as OA_Low_ following stimulation for all three eGFP-FoxO proteins (representative images in Figure 3C, quantification in Figure 3D). Importantly, transient expression of ARPP19 and FAM122 resulted in partial inhibition of eGFP-FoxO1 nuclear translocation following stimulation but had no effect on eGFP-FoxO3a or -FoxO4 (representative images in Figure 3C; quantification in Figure 3D).

To further confirm these observations, we depleted *B55* and *B56* mRNA utilizing previously validated shRNA constructs [26,28]. Because HeLa cells are deficient for expression of select B55 and B56 proteins, we focused our analyses on evaluating the most abundantly expressed family members. Transient expression of knockdown constructs in HeLa cells resulted in robust depletion of target *B55* and *B56* mRNAs 72 h post-transfection (Figure 3E). Knockdown constructs were transiently expressed in eGFP-FoxO stables cells for 72 h prior to PI3K_i_ treatment and evaluation of nuclear translocation efficiency. As anticipated, knockdown of *B55A* resulted in nuclear translocation inhibition for eGFP-FoxO1 following stimulation but had no impact on eGFP-FoxO3a or -FoxO4 nuclear translocation. Moreover, inhibition of eGFP-FoxO1 nuclear translocation in *B55A* knockdown cells was equivalent to the inhibition observed following OA_Low_ treatment, which supports observations above that eGFP-FoxO1 is regulated by a PP2A-B55 subcomplex. Likewise, combinatorial transient expression of *B56* knockdown constructs resulted in eGFP-FoxO3a nuclear translocation inhibition equivalent to OA_Low_ treatment following stimulation but had no impact on eGFP-FoxO1 or -FoxO4 nuclear translocation (Figure 3G). Moreover, knockdown of single or dual *B56* mRNAs had no effect on eGFP-FoxO3a nuclear translocation, suggesting that multiple distinct PP2A-B56 subcomplexes contribute to FoxO3a regulation. Interestingly, eGFP-FoxO4 was not affected by functional inactivation of PP2A-B55 or -B56 subcomplexes yet is sensitive to OA treatment.

### 3.4. Altering the FoxO3a B56-Interacting Motif Modulates Nuclear Translocation Kinetics

As mentioned above, B56 regulators engage cellular substrates through a highly conserved LxxIxE motif [47]. Biochemical studies have established a hierarchy of substrate binding affinities with a preference for leucine > methionine > phenylalanine at the first position, isoleucine > valine > leucine at the fourth position, and an exclusive preference for glutamate at the terminal position. Furthermore, additional glutamate residues following the terminal glutamate can strongly enhance binding affinity [47].

**Figure 4:**
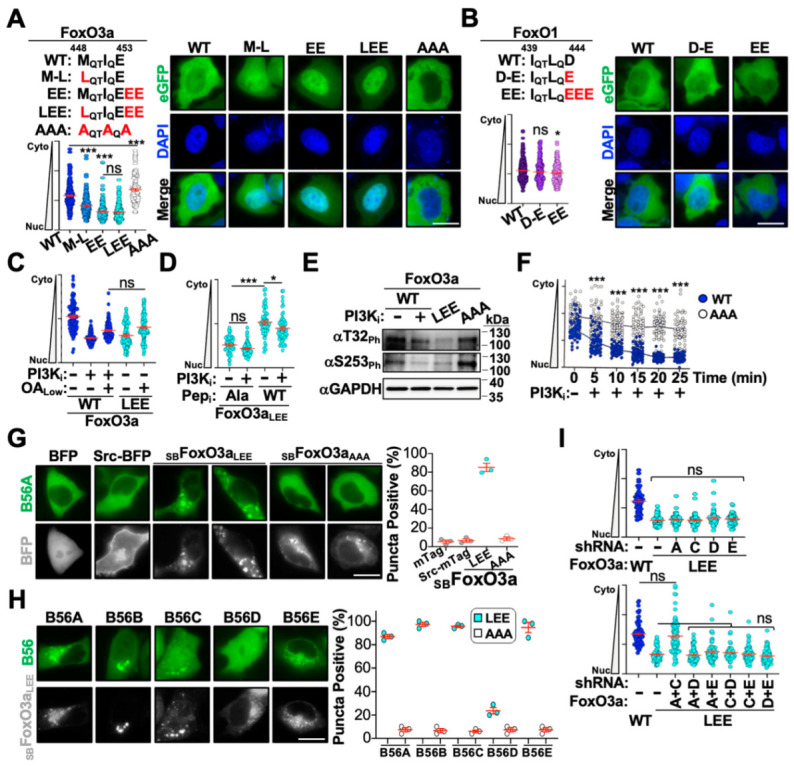
Defining FoxO3a-PP2A-B56 interaction dynamics. (**A**) Functional analysis of FoxO3a B56-motif mutants. Wild-type sequence depicted in black with mutated residues depicted in red. Representative fluorescence microscopy images of the indicated eGFP-FoxO3a protein shown on right, quantification of localization shown on bottom (n = 75). (**B**) Functional analysis of FoxO1 B56-motif mutants. Wild-type sequence depicted in black with mutated residues depicted in red. Representative fluorescence microscopy images of the indicated eGFP-FoxO3a protein shown on right, quantification of localization shown on bottom (n = 75). (**C**) Quantification of cytoplasm-to-nucleus localization of eGFP-FoxO3a wild-type and LEE stable cell lines treated with OA_Low_ in the presence and absence of stimulation (n = 75). (**D**) Quantification of cytoplasm-to-nucleus localization of eGFP-FoxO3a_LEE_ stable cell lines transiently expressing wild-type and alanine B56-peptide inhibitors following PI3K_i_ treatment (n = 75). (**E**) Immunoblot analysis of phosphorylation at two key residues in eGFP-FoxO3a wild-type, LEE, and AAA stable cell lines in the presence or absence of PI3K_i_ treatment. (**F**) Quantification of cytoplasm-to-nucleus translocation of eGFP-FoxO3a wild-type and AAA stable cells following PI3K_i_ treatment for the indicated time (n = 60). (**G**,**H**) Representative images and quantification of puncta formation in cells transiently expressing Src-tagged FoxO proteins and eGFP-tagged B56 proteins. At least 50 cells were analyzed per biological replicate, each replicate is depicted on right. (**I**) Quantification of cytoplasm-to-nucleus translocation of eGFP-FoxO3a wild-type and LEE stable cells transiently expressing control or the indicated *B56* knockdown constructs (n = 50). Scale bar = 10 μm; ns, not significant; *, *p* < 0.05; ***, *p* < 0.001.

With these constraints in mind, we looked for putative B56-binding motifs present in FoxO3a but deficient in the other FoxO family members. We identified a single putative M_QT_I_Q_E motif in FoxO3a which was either sufficiently different or deleted in other FoxO family members (Figure 1A and Figure 4A,B). To determine the role this motif plays in regulating eGFP-FoxO3a nuclear translocation, we mutated key motif residues to alter B56 binding affinity and assessed localization patterns in the absence of stimulation (Figure 4A). Substituting the methionine with leucine resulted in whole cell distribution under unstimulated conditions, while adding additional glutamate residues resulted in complete nuclear accumulation in the absence of stimulation (Figure 4A). As expected, substituting key motif residues with alanine resulted in significant cytoplasmic enrichment of eGFP-FoxO3a (Figure 4A). Interestingly, generating similar substitution mutations in the analogous FoxO1 B56-motif had no impact on localization under unstimulated conditions, suggesting that the motif alone is not sufficient for driving B56 interactions (Figure 4B). Next, we wanted to confirm that nuclear enrichment of the eGFP-FoxO3a_LEE_ mutant was due to enhanced interactions with B56 proteins. First, we assessed eGFP-FoxO3a_LEE_ responsiveness to PP2A inhibition. Treating eGFP-FoxO3a_LEE_ stable cells with OA_Low_ induced partial redistribution from the nucleus to the cytoplasm at an abundance similar to that of eGFP-FoxO3a stable cells treated with OA_Low_ and stimulated with PI3K_i_ (Figure 4C). Likewise, transient expression of the B56-peptide inhibitor diminished nuclear accumulation of eGFP-FoxO3a_LEE_ in the presence and absence of stimulation, further indicating that this mutant enhances interactions with a PP2A-B56 complex (Figure 4D). In addition, these eGFP-FoxO3a_LEE_ activities correlated with increased dephosphorylation of key regulatory residues known to influence cytoplasmic retention and with delayed nuclear translocation kinetics of the eGFP-FoxO3a_AAA_ mutant following stimulation (Figure 4E,F). Taken together, these findings strongly support a model wherein eGFP-FoxO3a is regulated by a PP2A-B56 complex through the M_QT_I_Q_E motif.

We next wanted to further define the PP2A-B56 subcomplexes that regulate eGFP-FoxO3a nuclear translocation. To test this, we developed an *in cellulo* co-immunoprecipitation-like technique that leverages relocalization of FoxO proteins to intracellular membranes to induce concomitant relocalization of B56 interactors. To achieve this, we fused the Src kinase membrane targeting domain to BFP-FoxO (_SB_FoxO) proteins to induce relocalization to endosomal membranes (Figure 4G).

Co-expression of eGFP-B56A with _SB_FoxO3a_LEE_ resulted in redistribution of eGFP-B56A to _SB_FoxO3a_LEE_ puncta, whereas co-expression with BFP, Src-BFP, or _SB_FoxO3a_AAA_ had no impact on eGFP-B56A localization (i.e., no puncta were observed) (Figure 4G). Interestingly, all B56 family member exhibited redistribution to _SB_FoxO3a_LEE_ puncta except for B56D, which was largely unresponsive to _SB_FoxO3a_LEE_ expression (Figure 4H). These observations suggested that PP2A-B56A, -B56C, and -B56E subcomplexes regulate FoxO3a nuclear translocation (B56B is not expressed in HeLa cells [26]). To confirm this, we transiently expressed *B56* knockdown constructs in eGFP-FoxO3a_LEE_ stable cells and assessed localization in the absence of stimulation (i.e., restoration of cytoplasmic localization). As noted above, individual knockdown of B56 proteins had no impact on nuclear accumulation of eGFP-FoxO3a_LEE_; however, when B56A and B56C were concomitantly depleted we observed strong relocalization of eGFP-FoxO3a_LEE_ from the nucleus to the cytoplasm (Figure 4I). Taken together, these findings support our previous observations above that eGFP-FoxO3a is regulated by multiple PP2A-B56 subcomplexes.

## 4. Discussion

While the signaling pathways and post-translational modifications that promote FoxO cytoplasmic retention have been studied extensively, the mechanisms regulating nuclear translocation and activation of transcriptional programs are less clear. Here, we demonstrate that PP2A regulates the cytoplasm-to-nucleus transition of FoxO1, FoxO3a, and FoxO4 proteins following inhibition of metabolic signaling or the induction of oxidative stress. Moreover, PP2A and AMPK coordinate to fine-tune FoxO nuclear translocation through inhibition of nuclear export as opposed to enhancing nuclear import. Using functional, genetic, and biochemical approaches we define the PP2A subcomplexes that regulate nuclear translocation of FoxO1 and FoxO3a proteins. Mutational analysis revealed that the FoxO3a-B56 interacting motif is necessary but not sufficient for promoting FoxO-B56 interactions as mutating the analogous motif on FoxO1 had no impact on localization. Introducing mutations into the FoxO3a-B56 interface to strengthen or weaken PP2A interactions affected nuclear translocation kinetics accordingly. Together, these studies shed light on the mechanisms that regulate FoxO nuclear translocation.

Accumulating evidence indicates that dysregulation of FoxO proteins is a major driver for the progression of several diseases including metabolic disorders, neurodegeneration, auto-immunity, and carcinogenesis [60,61,62,63]. Therefore, delineating the regulatory mechanisms that fine-tune FoxO activity under diverse cellular conditions is critical for defining their role in promoting disease progression. Likewise, identifying pathways that differentially regulate FoxO family members is equally as important. Genetic, pharmacologic, and biochemical experiments discussed above confirm that FoxO1, FoxO3a, and FoxO4 are all regulated by PP2A, albeit through specific mechanisms. While all three FoxO proteins exhibited sensitivity to PP2A inhibition, only FoxO1 and FoxO3a were responsive to loss of PP2A-B55 or -B56 subcomplexes, respectively. Mutagenesis and *in cellulo* co-immunoprecipitation experiments confirmed that FoxO3a is regulated by PP2A-B56 subcomplexes whereas FoxO1 is not. Interestingly, over-expression or genetic depletion (i.e., mRNA knockdown) of B56D had no impact on FoxO3a nuclear translocation whereas this was not the case for other B56 family members. Recent structural evidence suggests that B56D has an auto-inhibitory mechanism wherein N- and C-terminal long disordered arms occlude the substrate binding groove under steady-state conditions [64]. This provides a mechanistic rationale as to why FoxO3a nuclear translocation is refractory to B56D protein expression in knockdown and *in cellulo* co-immunoprecipitation experiments. Taken together, these observations uncover specific cellular mechanisms that differentially regulate FoxO proteins. Further studies will be necessary to define the FoxO1-B55 binding interface and to identify the PP2A subcomplexes that regulate FoxO4 nuclear translocation.

The PI3K/AKT/PTEN signaling axis is one of the most frequently mutated oncogenic pathways in cancer [65,66]. Dysregulation of this signaling cascade causes hyperactive AKT signaling and the constitutive phosphorylation of downstream targets, such as FoxO proteins. This induces the cytoplasmic sequestration of FoxO proteins and the inhibition of pro-apoptotic transcriptional programs. However, one important question that remains to be addressed experimentally is the subcellular location of FoxO phosphorylation and dephosphorylation. Emerging evidence indicates that AKT can translocate to the nucleus to regulate a wide array of cellular processes [42,43,44]. This raises the possibility that AKT phosphorylates FoxO proteins in the nucleus, as opposed to the cytoplasm, which induces 14-3-3 engagement and promotes nuclear export and cytoplasmic retention. In fact, two previous studies have suggested as much by demonstrating that nuclear export of FoxO1 can be modulated by altering AKT enzymatic activity or subcellular localization [67,68]. This mechanism also rationalizes our observations that leptomycin B treatment induces robust nuclear accumulation of FoxO proteins, and how leptomycin B can “override” PP2A and AMPK inhibition. Nevertheless, an appealing therapeutic strategy is to restore FoxO function in cancer by leveraging the induction of PP2A activity through small molecule activating compounds [69,70,71]. However, PP2A is also frequently inactivated in cancer through the upregulation of negative regulatory factors, downmodulation of PP2A-activating enzymes, functional inactivation of regulatory proteins, or germline inactivation of PP2A components [72,73,74]. Therefore, other therapeutic strategies will be required to harness FoxO-directed cell cycle regulation and pro-apoptotic responses. Here, we identify two novel biochemical approaches that could be leveraged to promote FoxO nuclear accumulation when AKT activity is high. First, we demonstrate that FoxO proteins undergo constitutive cytoplasm-to-nucleus shuttling that can be disrupted by the inhibition of nuclear export. Second, we demonstrate that mutating the FoxO3a-B56 interacting motif to increase binding affinity results in constitutive nuclear accumulation of FoxO3a proteins. Therefore, leveraging gene editing technologies to disrupt the FoxO nuclear export sequence or to knock-in high affinity B56-binding motifs could restore FoxO accumulation in the nucleus and the concomitant activation of transcriptional programs. However, more work needs to be carried out to further evaluate the mechanism underlying nuclear accumulation of the FoxO3a_LEE_ mutant. If this mutant is indeed engaging B56 proteins at higher affinity, it is possible that B56 binding is occluding AKT access or 14-3-3 engagement. Future dedicated studies will be required to determine if this approach can be utilized to restore FoxO function and slow tumor progression in vitro and in vivo.

## 5. Conclusions

We demonstrate that PP2A and AMPK combine to regulate the nuclear accumulation of several FoxO family members following inhibition of metabolic signaling or the induction of oxidative stress. Moreover, we determine that PP2A subcomplexes containing B55 and B56 proteins regulator the nuclear accumulation of FoxO1 and FoxO3a proteins, respectively. Mutating a putative B56-FoxO3a interacting motif to enhance or diminish PP2A binding affinity altered nuclear translocation kinetics accordingly. Taken together, these observations reveal novel mechanistic insights into the regulation of FoxO transcription factors.

## Data Availability

All original contributions presented in this study are included in the article and Supplementary Materials. Further inquiries can be directed to the corresponding author (salamango@uthscsa.edu).

## Supplementary Materials

The following supporting information can be downloaded at: https://www.mdpi.com/article/10.3390/cells14050342/s1, Figure S1: Uncropped immunoblot images from Figure 4E.
