# Supplementary material for: Defining the Protein Phosphatase 2A (PP2A) Subcomplexes That Regulate FoxO Transcription Factor Localization
Source: Cells. 2025 Feb 27;14(5):342. doi: 10.3390/cells14050342 (PMC11899004; doi:10.3390/cells14050342)
Supplement: Supplementary file 1 [file cells-14-00342-s001.zip › cells-3479739-supplementary.pdf]

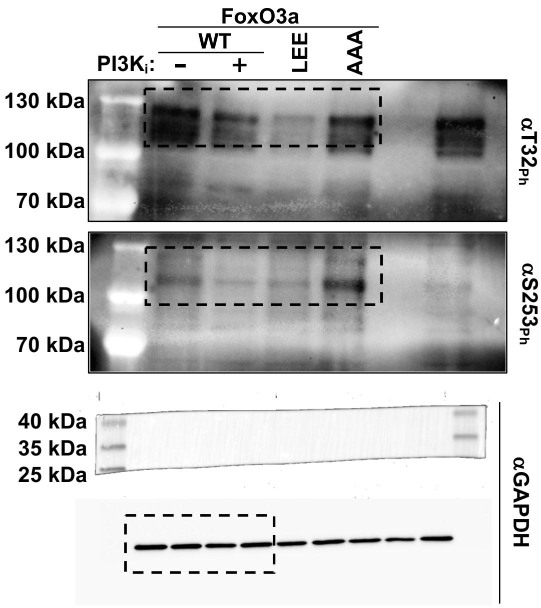

FIGURE S1. Uncropped immunoblot images from Figure 4E. Sample well are labeled as follows, lane 1 is the protein ladder, lane 2 is the wild-type eGFP-FoxO stable cell line in the absence of treatment, lane 3 is the wild-type eGFP-FoxO stable cell line following $PI3K_i$ treatment, lane 4 is the untreated $eGFP\text{-}FoxO_{LEE}$ stable cell line, and lane 5 is the untreated $eGFP\text{-}FoxO_{AAA}$ stable cell line. Size markers are depicted on the left while the antibodies against the indicated cellular targets are depicted on the right. The dashed boxes indicate the region of the blots shown in Figure 4E.
